# Bookreview of principles of data integration

**DOI:** 10.3389/fninf.2013.00011

**Published:** 2013-06-18

**Authors:** Martin Telefont

**Affiliations:** Blue Brain Project, École Polytechnique Fédérale de LausanneLausanne, Vaud, Switzerland

Large-scale data gathering efforts of the past, like the Human Genome Project, have shown that their most valuable contribution is data, which allows researchers to link their own experimental findings to them. This process, data integration, will be of central importance in how the scientific community to will be able to draw on the result of the next decade which doubtless will be the decade of the brain, in live sciences. Doan et al. (2012) introduce the current state of Data Integration to a general, academically trained, readership. The topics are split into nineteen chapters. After an initial introductory chapter, the remainder of the book is split into three parts, “Foundational Data Integration Techniques,” “Integration with Extended Data Representations,” and “Novel Integration Architecture.” Through the book the authors are leading the novice from basic concepts to the current state of data integration principles and techniques.

Examples of data integration problems are taken from a range of easily accessible situations. The most extensive examples focus on business and movies centric data. However, the range of less extensive examples show how the discussed processes are applicable to a wide range of situations.

The first introductory chapter introduces the reader to the world and challenges of data integration. The authors use diagrams and illustrations of concepts and approaches to show the reader a map, which serves him well in seeing how subsequent chapters fit into the larger domain of Data Integration. Some passages could have been streamlined to communicate the basics more explicitly. The great number of examples are however likely to be helpful to readers of non-technical backgrounds.

The first section of the book (chapters 2–10) is dedicated to “Foundational Data Integration Techniques.” In this section, the authors take the reader from data organization in classical, database driven setups to use-cases, which are commonplace in data integration. This allows the reader to become familiar with basic concepts in data access, storage, and integration. The authors do not offer product or application centered tutorials but lead the reader into a discussion that explains how different solutions work and their respective drawbacks. Sometimes the transition from introduction to the formula rich explanation style feels abrupt. The description of these algorithmic procedures is short, crisp and to the point.

With the exception of the last two chapters, “Wrappers,” and “Data Warehousing and Caching,” this section of the book is procedure centric. Providing a glimpse of the complexity underlying topics that casual technology users often take for granted. By the end of the section, the reader has the impression she is ready to go to the primary literature for more detailed information.

The last two chapters go in to some details why data integration should not be practiced by looking for a “one size fits all” approach but be customized to the problem at hand.

Section 2, “Integration with Extended Data Representations” confronts the reader with terms, which are part of today's computational reality. While most readers have come across XML, perhaps fewer are aware of DTD, XSD, XQuery, and XPath. As in the previous section diagrams and illustrations provide focus, enormously facilitating the communication of key concepts and keeping the reader engaged in what would otherwise be a dry technical discussion.

In the past ten years, many academic domains have used ontologies and other forms of knowledge representations to capture domain knowledge. Chapter 12 introduces how these concepts are useful in the context of data integration. While the chapter is nicely done it will be more accessible to people working in domains where the use of ontologies are less novel. The introduction of standards like RDF and OWL provide readers with a good starting point for seeing how they can best apply to the different settings people conduct their work in.

In data integration a key theme is uncertainty when integrating information from multiple sources or measurement techniques. The authors do a decent job in introducing ideas and providing examples, but one cannot help but feel that they could have offered a more extensive treatment. The authors show how one is able to use a probabilistic approach on how to address the uncertainty in the mapping between data sources and across different information modalities. It would have more informative if a number of different approaches would have been contrasted on how they address the same problem.

Data Provenance is often dismissed as something, which can be taken care off by adding an extra field to a database table. The authors do an excellent job of explaining why this is not enough. Being able to backtrack the process of information generation is vital to maintaining an integration tool chain that can be improved. Without this results of complex operations are more likely to result in interesting outcomes not all of which are explainable in a simple way. This topic is often under-appreciated by non-practitioners but essential in a reliable production environment.

The last part of the book discusses new phenomena resulting for the gradual emergence of Web 2.0. The chapters on “Peer-to-Peer Integration” and “Integration for Collaboration” provide insight into the directions Data Integration is likely to take in coming years. After the previous methods-heavy chapters the discussion allows the reader to look at common services and methods through new eyes.

In summary the authors have achieved something which is rare in academic books on technology. They balance a popular account of research with enough technical vocabulary and understanding for readers to engage with the research community. Only rarely have I read book that is so effective in introducing the complexity of a new topic, introducing readers to new methods, and describing emergent trends. Although the learning curve is steeper for some chapters than for others, “new material” is nicely balanced by more familiar topics. After reading the book a reader is not fluent in methods of data integration but he has acquired enough of a vocabulary and a perspective to continue the journey on his own.
